# Supplementary material for: ATP13A2 Loss of Function-Driven Polyamine Dysregulation Induces SAM Depletion and Epigenetic Astrocyte Toxicity
Source: bioRxiv. 2026 Apr 6:2026.04.02.716164. Preprint. [Version 1] doi: 10.64898/2026.04.02.716164 (PMC13081846; doi:10.64898/2026.04.02.716164)
Supplement: Supplement 1 [file media-1.pdf]

Key Resource Table

Resource Type	Resource Name	Source	Identifier	New/Reuse	Additional info
**Dataset**	bulkRNA-seq data from WT, ATP13A2 c.1306	This paper	https://doi.org/10.5281/zenodo.19226018	new	
**Dataset**	bulkATAC-seq data from WT, ATP13A2 c.1306	This paper	https://doi.org/10.5281/zenodo.19226012	new	
**Dataset**	bisulfite sequencing	This paper	https://doi.org/10.5281/zenodo.19226020	new	
**code**	bulkRNA-seq data from WT, ATP13A2 c.1306	This paper	https://github.com/blanchardlab/ATP13A2-paper/tree/main/RNAseq	new	
**code**	bulkATAC-seq data from WT, ATP13A2 c.1306	This paper	https://github.com/blanchardlab/ATP13A2-paper/tree/main/ATACseq	new	
**code**	bisulfite sequencing	This paper	https://github.com/blanchardlab/ATP13A2-paper/tree/main/Bisulphateseq	new	
**Experimental model: Organism/strain**	Sox2-cre expressing female mice	This paper	RRID:IMSR_JAX:008454	reuse	
**Experimental model: Organism/strain**	Atp13a2 Flox male mice	This paper	RRID:IMSR_JAX:028387	reuse	
**Experimental model: iPSC**	KOLF2.1 J (ATP13A2WT)	Jax Laboratory	RRID:CVCL_B5P3	reuse	
**Experimental model: iPSC**	KOLF2.1J c1306 SNV/SNV	Jax Laboratory	RRID:CVCL_F2AU	reuse	
**Protocol**	Brain tissue collection	protocols.io	dx.doi.org/10.17504/protocols.io.261gedq8ov47/v1	reuse	
**Protocol**	Perfusion	protocols.io	dx.doi.org/10.17504/protocols.io.5jyl8p3qrg2w/v1	reuse	
**Protocol**	Brain sectioning using vibratome	protocols.io	dx.doi.org/10.17504/protocols.io.j8nlko72xv5r/v1	reuse	
**Protocol**	Immunofluorescent staining	protocols.io	dx.doi.org/10.17504/protocols.io.3byl4qoxrvo5/v1	reuse	
**Protocol**	iPSC cultures	protocols.io	10.17504/protocols.io.eq2ly523pvx9/v1	new	
**Protocol**	Midbrain organoids differentiation	protocols.io	dx.doi.org/10.17504/protocols.io.rm7vzbnr4vx1/v1	reuse	
**Protocol**	Midbrain neuronal culture	protocols.io	10.17504/protocols.io.x54v9bzmml3e/v1	new	
**Protocol**	NGN2 neuronal culture	protocols.io	10.17504/protocols.io.j8nlk12ewg5r/v1	new	
**Protocol**	Midbrain Astrocytes extraction and culture	protocols.io	dx.doi.org/10.17504/protocols.io.261ge364wl47/v2	new	
**Protocol**	Co-cultures	protocols.io	10.17504/protocols.io.rm7vze28xvx1/v1	new	
**Protocol**	Conditioned Media Experiments	protocols.io	10.17504/protocols.io.8epv55m1nv1b/v1	new	
**Protocol**	Protein Extraction and Immunoblotting	protocols.io	dx.doi.org/10.17504/protocols.io.q26g7y428gwz/v1	new	
**Protocol**	Lentiviral Production and Transduction	protocols.io	dx.doi.org/10.17504/protocols.io.6qpvr8ydolmk/v1	reuse	
**Protocol**	Live-Cell imaging of α-synuclein internalization	protocols.io	10.17504/protocols.io.261ge1jowv47/v1	reuse	
**Protocol**	RNA Extraction and RT-qPCR	protocols.io	dx.doi.org/10.17504/protocols.io.3byl414krlo5/v1	reuse	
**Protocol**	Immunofluorescence and Confocal Microscopy	protocols.io	dx.doi.org/10.17504/protocols.io.eq2lyqmemvx9/v1	reuse	
**Protocol**	Effector Caspase assay	protocols.io	dx.doi.org/10.17504/protocols.io.6qpvrqb5blmk/v1	reuse	
**Protocol**	LDH Cytotoxicity Assay	protocols.io	10.17504/protocols.io.q26g776b3gwz/v1	new	
**Protocol**	Lysosomal Function and Phagocytosis Assays	protocols.io	dx.doi.org/10.17504/protocols.io.14egn2xmyg5d/v1	reuse	
**Protocol**	Cytokine Profiling	protocols.io	10.17504/protocols.io.kxygx82eov8j/v1	new	
**Protocol**	Polyamine Uptake Assay - FACS	protocols.io	10.17504/protocols.io.q26g7mpq1gwz/v1	new	
**Protocol**	Polyamine Uptake Assay - Confocal imaging	protocols.io	10.17504/protocols.io.5qpvo9kd9v4o/v1	new	
**Protocol**	Dotblot aggregated a-synuclein	protocols.io	dx.doi.org/10.17504/protocols.io.bp2l629x1gqe/v1	new	
**Protocol**	Astrocytes Viral Transfection	protocols.io	dx.doi.org/10.17504/protocols.io.n2bvjbwxpgk5/v1	new	
**Antibody**	α-synuclein	Abcam	#AB138501	reuse	1:300 staining, 1:1000 blots
**Antibody**	Actin	EMD Millipore	#S2532	reuse	1:2000
**Antibody**	Aggregated-synuclein	Abcam	#AB209538	reuse	1:1000
**Antibody**	ATP13A2	Sigma	#A3361	reuse	1:1000
**Antibody**	CD44	Cell Signaling Technology	#3570S	reuse	1:1000
**Antibody**	FOXA2	Abcam	#AB60721	reuse	1:500
**Antibody**	GAPDH	Abcam	#ab9485	reuse	1:1000
**Antibody**	GFAP	EMD millipore	#AB5804	reuse	1:300
**Antibody**	GFAP	Dako	#AB_10013382	reuse	1:300
**Antibody**	GIRK2	Alomone labs	#APC-006	reuse	1:300
**Antibody**	Histone 3 K9 dimethylation	Abcam	#AB_449854	reuse	1:300
**Antibody**	Histone 3 K4 trimethylation	Abcam	#AB_306649	reuse	1:300
**Antibody**	LAMP1	Novus biotech	#NBP2-25183	reuse	1:300
**Antibody**	MAP2	BioLegend	#822501	reuse	1:500
**Antibody**	S100A6	Abcam	#ab181975	reuse	1:200
**Antibody**	S100B	EMD millipore	#S2532	reuse	1:300
**Antibody**	SOX9	Abcam	#AB185966	reuse	1:1000
**Antibody**	TH	Abcam	#ab112	reuse	1:300
**Antibody**	TUJ1	BioLegend	#801202	reuse	1:300
**Chemical, peptide, or recombinant protein**	DAPI	Cayman Chemical	#13197	reuse	1:5000
**Chemical, peptide, or recombinant protein**	Recombinant aSynuclein-HiLyte Fluor 488 labeled	Anaspec	#AS-55457	reuse	
**Chemical, peptide, or recombinant protein**	CXCL1	Peprotech	#275-GR-010	reuse	
**Software/code**	FIJI Version 2.10.0	National Institute of Health (NIH)	https://imagej.net/software/fiji/; RRID: SCR_002285	reuse	
**Software/code**	Cell Profiler	Broad Institute	https://cellprofiler.org	reuse	
**Software/code**	GraphPad Prism Version 10.6.0		https://www.graphpad.com	reuse	
**Dataset**	Quantification data	Zenodo	10.5281/zenodo.18704033	new	
