# Supplementary material for: ATP13A2 Loss of Function-Driven Polyamine Dysregulation Induces SAM Depletion and Epigenetic Astrocyte Toxicity
Source: bioRxiv. 2026 Apr 6:2026.04.02.716164. Preprint. [Version 1] doi: 10.64898/2026.04.02.716164 (PMC13081846; doi:10.64898/2026.04.02.716164)

bioRxiv preprint doi: https://doi.org/10.64898/2026.04.02.716164; this version posted April 6, 2026. The copyright holder for this preprint (which was not certified by peer review) is the author/funder, who has granted bioRxiv a license to display the preprint in perpetuity. It is made available under aCC-BY 4.0 International license.

-

Figure S1. **a.** Sanger sequencing results of KOLF2.1J lines used for the project, which evidenced the single SNP G>A that causes the c.1306 mutation. **b.** Western blot of midbrain astrocytes of ATP13A2 and GAPDH as loading control and quantification of normalized levels of ATP13A2; differences analyzed by Two-tailed t-test. **c.** mRNA fold change by qPCR of midbrain organoids at d15 and d35 to corroborate expression of midbrain-specific markers. Undifferentiated iPSC value indicated by the undashed line (value=1). **d.** Micrography of TH (brown) and Hematoxylin (blue) of midbrain organoids at d25. Scale bar 20µm. Differences were analyzed by a two-tailed t-test. **e.** Micrography GIRK2 (brown) and Hematoxylin (blue) staining in $ATP13A2^{WT}$ and $ATP13A2^{c.1306}$ day 100 midbrain organoids and quantification. Scale bar 20µm. Differences were analyzed by a two-tailed t-test. **f.** Brightfield images of midbrain astrocytes in 2D culture. **g.** Immunocytochemistry of astrocytes stained for cell type (s100B, SOX9), and region (FOXA2) specific markers expression (green) and Hoechst (blue). Quantification of marker-positive cell percentage. Scale bar 50µm. **h.** Immunocytochemistry of mixed genetic co-cultures of midbrain neurons stained for MAP2 (green), midbrain astrocytes CD44 (magenta), and Hoechst (blue), and their quantification. Scale 50µm. **i.** Immunoblots of WT cortical neurons (NGN2) co-cultured with astrocytes for TUJ1 and GFAP. GAPDH was used as a loading control. Differences were analyzed by a two-tailed t-test.

bioRxiv preprint doi: https://doi.org/10.64898/2026.04.02.716164; this version posted April 6, 2026. The copyright holder for this preprint (which was not certified by peer review) is the author/funder, who has granted bioRxiv a license to display the preprint in perpetuity. It is made available under aCC-BY 4.0 International license.

Figure S2. **a.** ClusterProfiler dot plots of the GO terms for the ATP13A2$^{c.1306}$ vs ATP13A2^{WT} upregulated and downregulated genes detected in bulk RNAseq. **b.** mRNA fold change by qPCR of neuroinflammation-associated genes in ATP13A2^{WT} astrocytes treated with the PA transport inhibitor AMXT-1501, or control (DMSO). Differences were analyzed by Two-way ANOVA. **c.** DQBSA MFI and **d.** Lysosensor MFI of astrocytes treated with the PA transport inhibitor AMXT-1501, or control (DMSO). Differences were analyzed by a two-tailed t-test. **f.** Immunoblots of ATP13A2^{WT} and ATP13A2$^{c.1306}$ astrocytes for α-synuclein. GAPDH was used as a loading control. Differences were analyzed by a two-tailed t-test. **g.** mRNA fold change by qPCR of *SNCA*. differences analyzed by Two-tailed t-test **h.** Schematic of α-synuclein uptake experiments. Astrocytes transduced with mCherry-LAMP1 viruses were exposed to fluorescently labeled α-synuclein monomers. Live imaging over time allowed us to quantify overall total α-synuclein in cells and co-localizations with LAMP1-positive signal (lysosomes).

Figure S3 a. LDH assay in ATP13A2^{WT} exposed to astrocyte condition media at week one to three. **b.** ELISA for CXCL1 in conditioned media from astrocytes, differences analyzed by paired Two-tailed t-test. **c.** Immunofluorescence of TH (magenta), MAP2 (green), and active (cleaved) effector caspase 3/7 (blue) in neurons exposed to ATP13A2^{WT} conditioned media supplemented with 100ng/ml of recombinant CXCL1. Differences were analyzed by a two-tailed t-test. **d.** Quantification of MAP2/nuclei in neurons exposed to ATP13A2^{WT} conditioned media supplemented with increasing amounts of recombinant CXCL1 (Fig 3h and S3c). Differences were analyzed by One-way ANOVA.

Figure S4. a. Quantification of BODIPY-labeled and spermine (SPM) and spermidine (SPD) in astrocytes through median fluorescence intensity. Differences were analyzed by Two-way ANOVA. **b.** mRNA fold change by qPCR of *ODC1* and *AMD1*. Differences were analyzed by Two-way ANOVA. **c.** Volcano plot of differentially methylated promoters in ATP13A2^{c1306} vs ATP13A2^{WT} astrocytes. Labeled neuroinflammatory genes color-coded per methylation difference % **d.** Pie chart showing distribution of the differentially open peaks detected by ATAC-seq in ATP13A2^{WT} and ATP13A2^{c1306} astrocytes **e.** MA plot displaying peak accessibility in ATP13A2^{c1306} vs. ATP13A2^{WT} astrocytes. M (y-axis) denotes the log2fold change and A (x-axis) the mean of normalized counts. Individual peaks are color-coded by their annotated genomic regions. Bar plots summarize the percentage of DARs localized to specific genomic features **f.** Genomic snapshots of selected inflammatory signaling and astrocyte reactivity genes in ATP13A2^{WT} (blue) and ATP13A2^{c1306} (yellow) astrocytes. Tracks show normalized peak heights.

Figure S5. a. mRNA fold change by qPCR of *GFAP* in ATP13A2^{WT} and ATP13A2$^{c.1306}$ astrocytes treated with MGBG or control solution (H_2O). Differences were analyzed by One-way ANOVA. **b.** Median fluorescence intensity of DQ-BSA to measure proteolytic function in astrocytes targeted with shRNA for AMD1 or control shScramble. Differences were analyzed by One-way ANOVA. **h.** mRNA fold change by qPCR of *GFAP* in astrocytes targeted with shRNA for AMD1 shScramble. Differences were analyzed by One-way ANOVA.

bioRxiv preprint doi: https://doi.org/10.64898/2026.04.02.716164; this version posted April 6, 2026. The copyright holder for this preprint (which was not certified by peer review) is the author/funder, who has granted bioRxiv a license to display the preprint in perpetuity. It is made available under aCC-BY 4.0 International license.

Figure S1

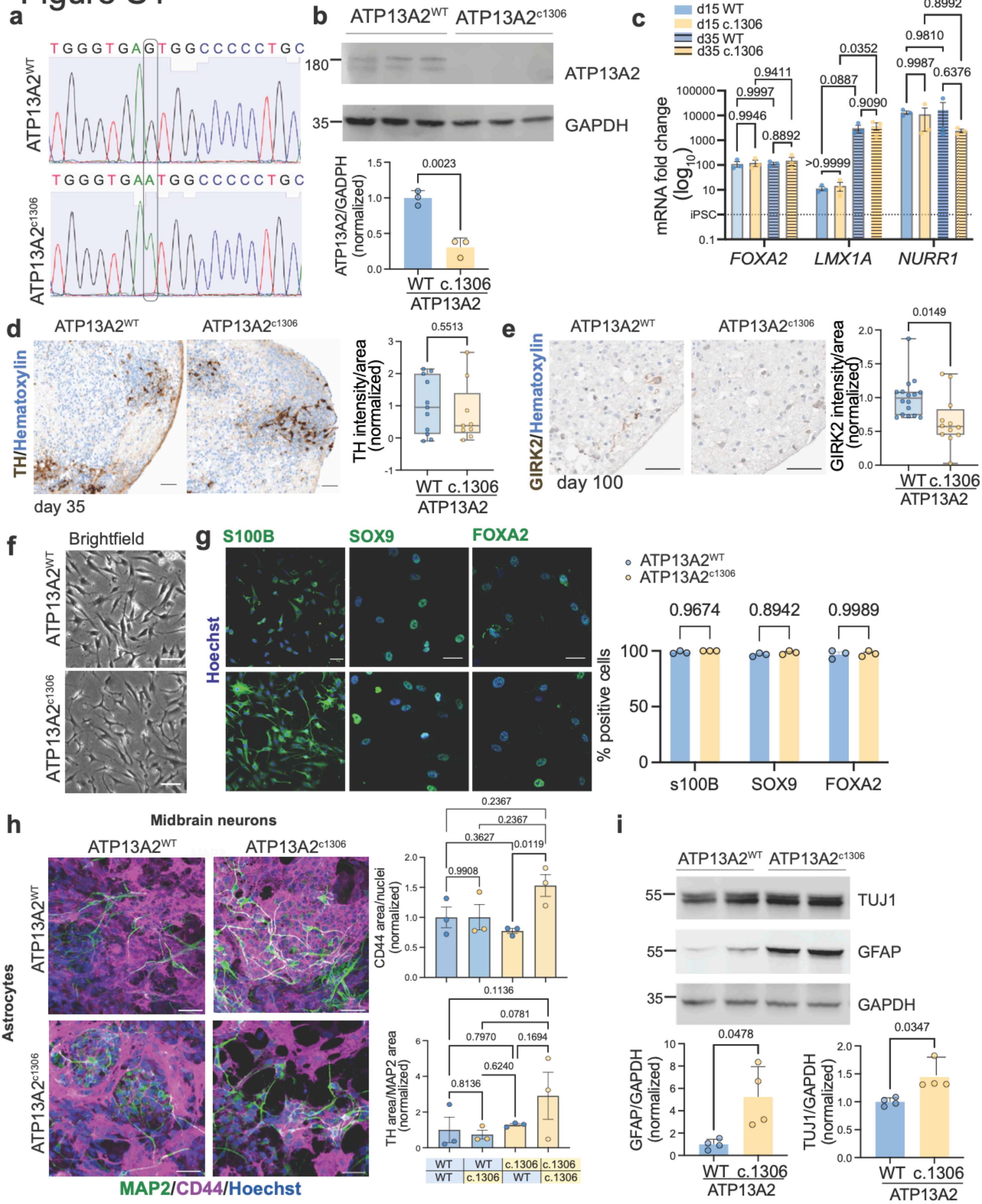

bioRxiv preprint doi: https://doi.org/10.64898/2026.04.02.716164; this version posted April 6, 2026. The copyright holder for this preprint (which was not certified by peer review) is the author/funder, who has granted bioRxiv a license to display the preprint in perpetuity. It is made available under aCC-BY 4.0 International license.

Figure S2

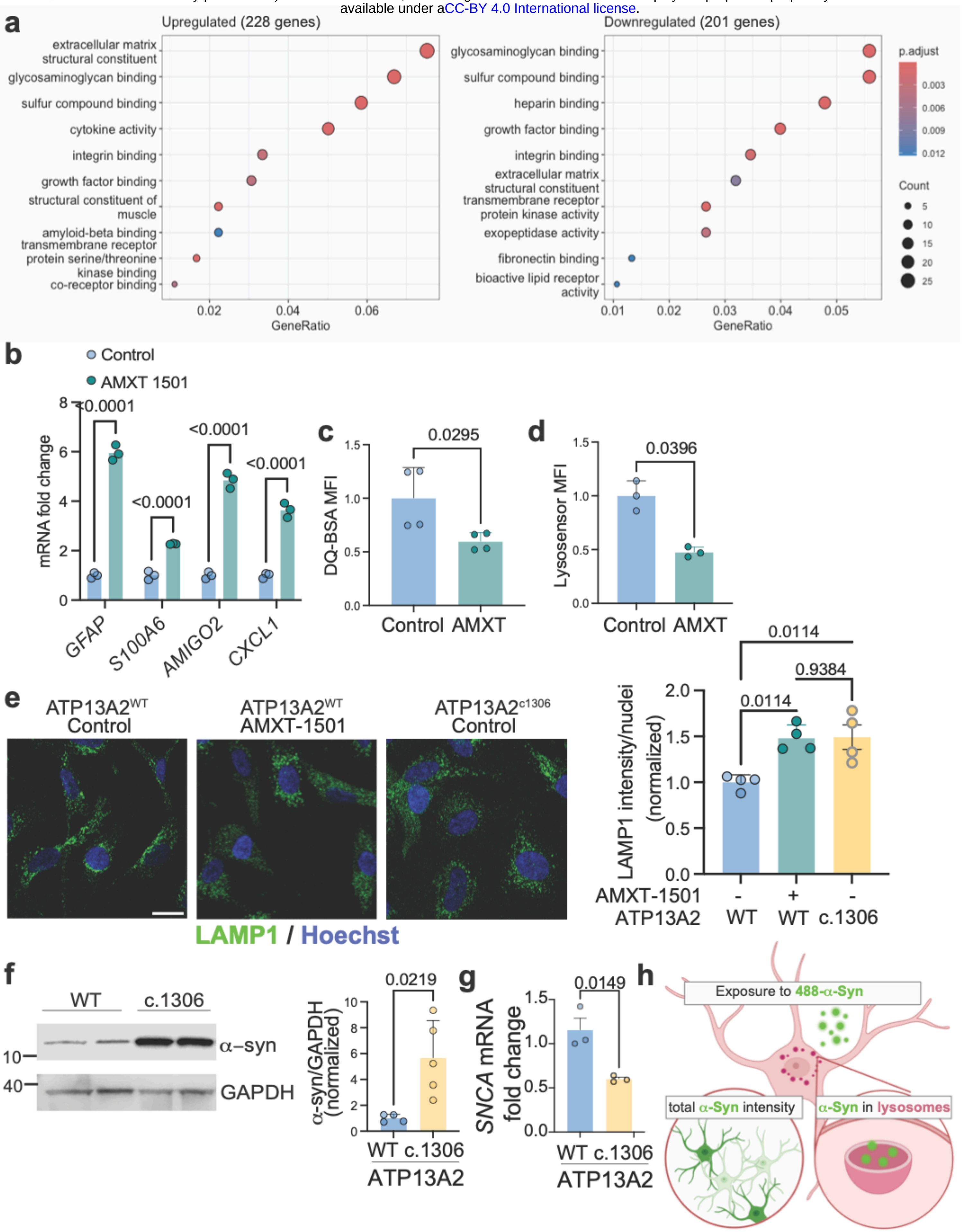

bioRxiv preprint doi: https://doi.org/10.64898/2026.04.02.716164; this version posted April 6, 2026. The copyright holder for this preprint (which was not certified by peer review) is the author/funder, who has granted bioRxiv a license to display the preprint in perpetuity. It is made available under aCC-BY 4.0 International license.

Figure S3

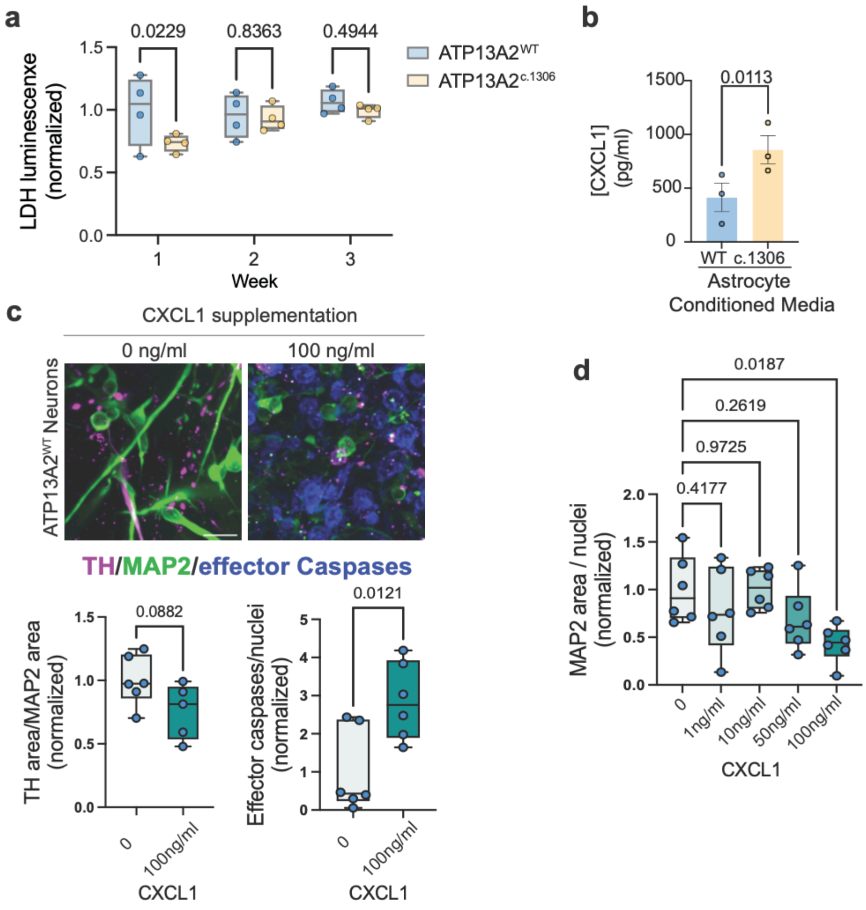

bioRxiv preprint doi: https://doi.org/10.64898/2026.04.02.716164; this version posted April 6, 2026. The copyright holder for this preprint (which was not certified by peer review) is the author/funder, who has granted bioRxiv a license to display the preprint in perpetuity. It is made available under aCC-BY 4.0 International license.

Figure S4

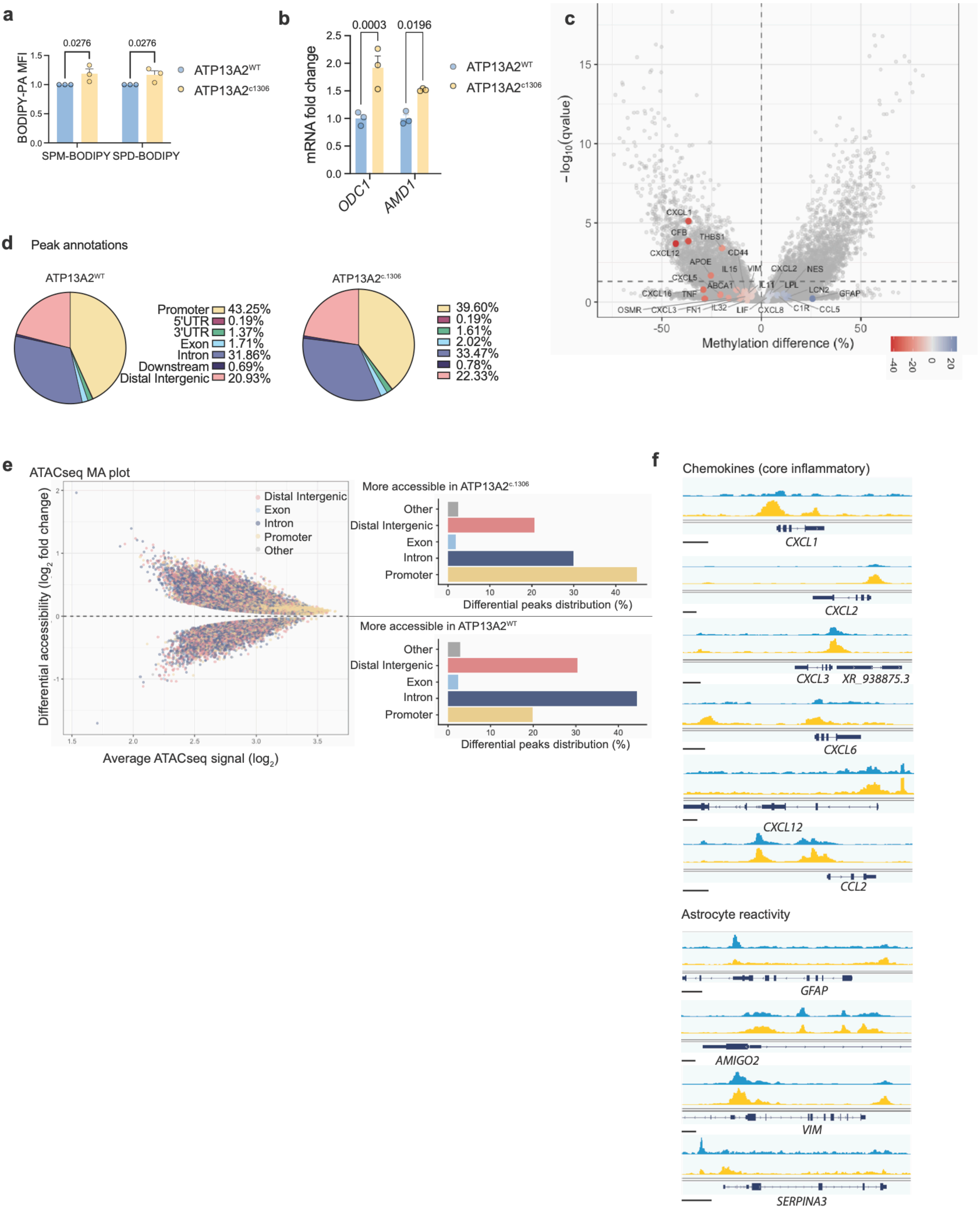

bioRxiv preprint doi: https://doi.org/10.64898/2026.04.02.716164; this version posted April 6, 2026. The copyright holder for this preprint (which was not certified by peer review) is the author/funder, who has granted bioRxiv a license to display the preprint in perpetuity. It is made available under aCC-BY 4.0 International license.

Figure S5

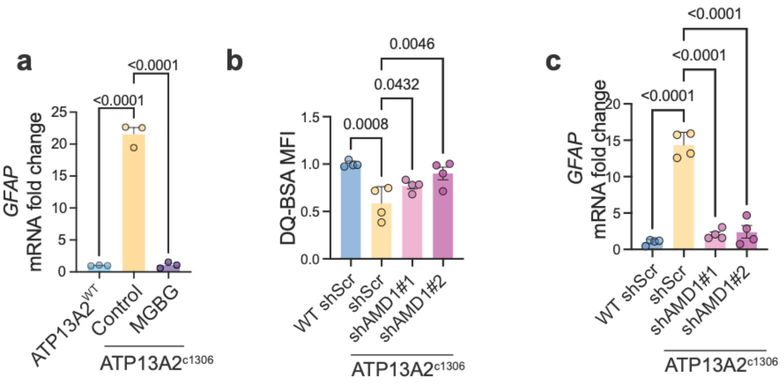

Supplement: 2 [file NIHPP2026.04.02.716164v1-supplement-2.pdf]
